# Management of bleeding in patients treated with direct oral anticoagulants

**DOI:** 10.1186/s13054-016-1413-3

**Published:** 2016-08-20

**Authors:** Marcel Levi

**Affiliations:** 1 Department of Vascular Medicine, Academic Medical Center (E-2), University of Amsterdam, Meibergdreef 9, 1105 AZ Amsterdam, The Netherlands; 2 Department of Medicine, University of Amsterdam, Amsterdam, The Netherlands

**Keywords:** Anticoagulants, Hemorrhage, Direct-acting oral anticoagulants, Dabigatran, Rivaroxaban, Apixaban, Edoxaban

## Abstract

**Background:**

Recently, a new generation of direct-acting oral anticoagulants (DOACs) with a greater specificity towards activated coagulation factors was introduced based on encouraging results for efficacy and safety in clinical studies. An initial limitation of these new drugs was the absence of an adequate strategy to reverse the effect if a bleeding event occurs or an urgent invasive procedure has to be carried out.

**Main text:**

Specific reversing agents for DOACs have become available, however, and are now evaluated in clinical studies. For the anti-factor Xa agents (rivaroxaban, apixaban, and edoxaban) a number of studies have shown that the administration of prothrombin complex concentrate resulted in a correction of the prolonged prothrombin time and restored depressed thrombin generation after rivaroxaban treatment in a controlled trial in healthy human subjects. In view of the relatively wide availability of prothrombin complex concentrates, this would be an interesting option if the results can be confirmed in patients on oral factor Xa inhibitors who present with bleeding complications. More specific reversal can be achieved with andexanet, a new agent currently in development that competitively binds to the anti-factor Xa agents. For the direct thrombin inhibitor dabigatran, the administration of prothrombin complex concentrates showed variable results in various volunteer trials and efficacy at relatively high doses in animal studies. Recently, a Fab fragment of a monoclonal antibody (idarucizumab) was shown to be an effective reversal agent for dabigatran in human studies.

**Conclusion:**

For the new generation of DOACs, several reversal strategies and specific antidotes are under evaluation, although most interventions need further evaluation in clinical trials.

## Background

Anticoagulants are frequently prescribed agents for the prevention and treatment of a myriad of cardiovascular conditions. Conventional anticoagulant agents, such as vitamin K antagonists (VKAs; warfarin, phenprocoumon, or coumadin) and heparin or low molecular weight (LMW) heparin, are increasingly replaced by direct oral anticoagulants (DOACs) directly inhibiting factor Xa (e.g., rivaroxaban, apixaban, or edoxaban) or factor IIa (e.g., dabigatran). This new generation of anticoagulants is termed novel oral anticoagulants (NOACs) or DOACs. A large number of clinical studies have shown that these agents can prevent or treat acute or chronic thromboembolic diseases [1].

The most important complication of treatment with anticoagulant agents is hemorrhage, which may be serious, may cause long-term debilitating disease, or may even be life-threatening [2]. Bleeding in a patient on anticoagulants may require specific (additional) management; if the bleeding situation is sufficiently severe, swift reversal of the anticoagulant effect of the anticoagulant agent may even be required. Depending on the clinical situation, including the site and/or the severity of the bleeding, this reversal may take place in a few hours, but in some cases immediate reversal is needed [3]. Generally, each (immediate) reversal of anticoagulant treatment needs also to take into consideration the indication for the antithrombotic agents. For example, the immediate interruption of anticoagulants in a patient with recent venous thromboembolism will markedly increase the short-term risk of recurrent venous thrombosis or pulmonary embolism. Likewise, in a patient with advanced heart disease and atrial fibrillation, interruption of anticoagulants may increase the risk of cerebral or systemic embolism. Each of these specific clinical settings requires a careful and balanced individual assessment of the benefits and dangers of reversing anticoagulants (and potential strategies to keep the period of reversal as brief as possible). Here we will briefly describe the epidemiology of bleeding complications due to anticoagulants and various strategies to reverse the anticoagulant effect of antithrombotic agents, focusing on the new generation of anticoagulants (DOACs).

### Relevance, incidence, and risk factors for bleeding in patients on anticoagulant treatment

The relevance of hemorrhagic complications in patients on anticoagulants is clearly demonstrated in a series of observational studies. In a large study of 34,146 patients with acute ischemic coronary syndromes, anticoagulant-associated bleeding was associated with a 5-fold increased risk of death during the first month and a 1.5-fold higher mortality between 30 days and 6 months [4]. Major hemorrhage was an independent predictor of mortality across all subgroups that were analyzed.

Currently, VKAs are still the most frequently used oral anticoagulant agents for long-term prevention and treatment of a wide range of cardiovascular diseases. In well-controlled patients in clinical trials, treatment with VKAs increases the risk of major bleeding by 0.5–1.0 %/year and increases the risk of intracranial hemorrhage by about 0.2–0.3 %/year [5]. However, in three real-life surveys this incidence varied from 1.4 to 3.4 %/year [6]. One needs to realize that the relatively uncomplicated trial populations may poorly reflect the real-life setting in which anticoagulants are prescribed. For example, in six pivotal trials that demonstrated the superiority of warfarin over placebo in the prevention of thromboembolic complications in patients with atrial fibrillation, 28,787 patients were screened but only 12.6 % of these patients were included in the studies [7].

The incidence of hemorrhagic complications with use of the new generation of oral anticoagulants with direct inhibitory properties towards thrombin or factor Xa is not very different. The severity and incidence of bleeding events in DOAC-treated patients has been evaluated in a number of clinical studies, which demonstrated similar or lower rates of major bleeding with DOACs compared with traditional anticoagulants [8–11]. The safety and efficacy of DOACs versus warfarin were evaluated in a meta-analysis of 14 prospective and retrospective studies in AF patients undergoing catheter ablation (4782 patients in total) [12]. Overall, no significant difference was observed in terms of thromboembolic events or major bleeding events between the warfarin and dabigatran treatment arms, although minor bleeding events occurred significantly less frequently with dabigatran compared with warfarin. Of note, DOAC treatment was associated with a reduced intracranial hemorrhage incidence compared with warfarin therapy, whereas some analyses showed an increased risk for gastrointestinal bleeds [12–14]. The recent guidelines from the European Society of Cardiology on anticoagulant management of atrial fibrillation recommend non-VKAs based on their more favorable incidence of hemorrhagic complications [15]. Likewise, DOACs are also advocated for the treatment of venous thromboembolism in recent guidelines [16]. Although these clinical study results are encouraging, there is very limited real-world evidence with regards to the incidence and severity of DOAC-related bleeding events; most of the currently available data are based on postmarketing studies.

Some clinical studies have reported on their experience with bleeding complications in patients treated with DOACs compared with patients on warfarin [17, 18]. Data from the EINSTEIN studies on rivaroxaban demonstrated that patients who presented with major bleeding on rivaroxaban had a relatively milder presentation and a better recovery compared with patients on warfarin. A prospective chart review of 15 emergency department patients treated with dabigatran and 123 patients treated with warfarin showed that patients on dabigatran had a shorter length of stay, fewer major bleeding events, and fewer life-threatening complications compared with patients on warfarin. Additionally, dabigatran use was more commonly associated with gastrointestinal hemorrhage, and less frequently associated with intracranial bleeding, compared with warfarin, in this patient population. This finding is in contrast to results from an analysis of insurance claim and administrative data from the FDA Mini-Sentinel database, which showed that dabigatran was associated with reduced incidence of gastrointestinal bleeds compared with warfarin [19]. The Australian Therapeutic Goods Administration has been monitoring the safety profile of dabigatran, and reported 361 serious bleeding events with this agent between 2011 and 2013 [20]. The most commonly reported bleeding site was the gastrointestinal tract, while a smaller proportion of intracranial bleeds were reported for dabigatran versus warfarin [20].

The most important risk factor for hemorrhage in users of anticoagulants is the intensity of the anticoagulant dose [21]. Studies suggest that with a target international normalized ratio (INR) of >3.0 the incidence of major bleeding is twice as large as in studies with a target INR of 2.0–3.0 [22]. In a meta-analysis of studies in patients with prosthetic heart valves, a lower INR target range resulted in a lower frequency of major bleeding and intracranial hemorrhage with a similar antithrombotic efficacy [23]. For dabigatran a clear relationship between dose and incidence of bleeding complications has been demonstrated in a clinical study in patients with atrial fibrillation [8], and all other DOACs have also demonstrated a similar dose-adverse event relationship [14].

Patient characteristics constitute another important determinant of the bleeding risk. Older patients have a 2-fold increased risk of bleeding [24], and the relative risk of intracranial hemorrhage (in particular at higher intensities of anticoagulation) was 2.5 (95 % CI 2.3–9.4) in patients aged >85 years compared with patients 70–74 years old [25]. Comorbidity may also significantly increase the risk of bleeding. A case–control study in 1986 patients on anticoagulants showed that comorbidity (such as mild renal insufficiency, hepatic dysfunction, or diabetes) increased the risk of bleeding by about 2.5 [7]. Another very important determinant of the risk of bleeding is the combined use of medication that affects both the coagulation system and platelet function. Two meta-analyses, comprising six trials with a total of 3874 patients and 10 trials with a total of 5938 patients, found a relative risk of major bleeding when antithrombotic agents were combined with aspirin of 2.4 (95 % CI 1.2–4.8) and 2.5 (95 % CI 1.7–3.7), respectively [26]. A population-based case–control study confirmed the high risk of upper gastrointestinal bleeding in patients using anticoagulants in combination with aspirin and/or clopidogrel [27]. It should be noted that the combined use of the new anticoagulant agents and antiplatelet agents was discouraged in the clinical trials, whereas this is increasingly common in clinical practice and may also have a serious impact on the risk of hemorrhage. Nonsteroidal anti-inflammatory agents (NSAIDs) are also associated with an enhanced risk of gastrointestinal bleeding. The combined use of anticoagulants and NSAIDs may result in an 11-fold higher risk of hospitalization for gastrointestinal bleeding as compared with the general population [28]. This risk is not significantly lower when using selective inhibitors of COX-2. As DOACs are associated with a higher risk of gastrointestinal bleeding compared with conventional anticoagulants (see above), the effect of combined treatment with these agents and aspirin or NSAIDs may be even larger.

### Reversal of DOACs

One of the main advantages of DOACs are the relatively stable pharmacokinetic and pharmacodynamic properties, which obviates the need for repeated control of the intensity of anticoagulation and dose adjustments. This means that for some clinical situations these drugs may represent an important improvement, but as already indicated the risk of (major) bleeding is still present. Clinical trials in patients using DOACs agents excluded many patients with common comorbidities and discouraged the simultaneous use of agents affecting platelet function. The risk of hemorrhage in these trials may therefore represent an underestimation of real-life bleeding risk.

Dependent on the severity of the clinical situation and in view of the relatively short half-life of the direct factor Xa inhibitors (5–15 h), cessation of medication may often be sufficient to reverse the anticoagulant effect in case of bleeding. Some authors argue that in most cases this will suffice and more immediate reversal is hardly ever really needed in clinical practice [29]. However, if immediate reversal of anticoagulation is deemed necessary, additional measures may be required. In general, these can be divided into nonspecific and specific interventions to reverse the anticoagulant effect (Table 1). A practical approach to the patient presenting with bleeding while using DOACs, based on recommendations of the European Heart Rhythm Association [15], is presented in Table 2.

**Table 1:** Pharmacological options for reversing the effect of the direct-acting oral anticoagulants

Inhibitor	Conventional dose
Nonspecific reversal (prohemostatic interventions)	
Prothrombin complex concentrates	50 U/kg
Activated prothrombin complex concentrates	50 U/kg
Recombinant factor VIIa	90 μg/kg
Specific reversal	
Directed at dabigatran	
Idarucizumab	5 g
Directed at rivaroxaban, apixaban, and edoxaban	
Andexanet-alfa	600–800 mg
Ciraparantag	100 mg

**Table 2:** Practical guide for how to manage bleeding complications in patients on direct oral anticoagulants

Oral thrombin inhibitors (dabigatran)	Oral factor Xa inhibitors (rivaroxaban, apixaban, edoxaban)
None life-threatening bleeding	
Check last intake; restoration of normal coagulation to be expected at 12–24 h (in case of creatinin clearance > 80 ml/min) or 24–36 h (in case of creatinin clearance 50–80 ml/min)	
Local hemostatic interventions, fluid management, transfusion	
Consider tranexamic acid (1000 mg 3dd) or DDAVP (0.3 μg/kg)	
Life-threatening bleeding	
All of the above	All of the above
Idarucizumab	Andexanet alfa
	Ciraparantag (under investigation)
Prothrombin complex concentrate (no evidence)	Prothrombin complex concentrate (healthy volunteer data)
Activated PCC (no evidence)	Activated PCC (no human evidence)
Recombinant factor VIIa (no evidence)	Recombinant factor VIIa (healthy volunteer data)

Suggested management strategy in case of hemorrhagic complications in patients using direct oral anticoagulants, modified from [15]

*PCC* prothrombin complex concentrate, *dd* daily, *DDAVP* de-amino D-arginine vasopressin

Nonspecific measures include (activated) prothrombin complex concentrates (PCCs) or recombinant factor VIIa (rFVIIa). The prothrombotic potential of activated PCCs and rFVIIa might be higher than that of nonactivated PCCs, so nonactivated PCCs may be preferred [30, 31]. In addition, a recent retrospective series of bleeding patients treated with PCCs for anticoagulant reversal showed a 20 % risk of thromboembolic complications, although part of the risk may have been due to the underlying thromboembolic risk for which the anticoagulant was prescribed in the first place and the clinical situation of the patients [32].

Specific measures are directly targeting the anticoagulant agent, by means of (Fab fragments of) monoclonal antibodies (in the case of dabigatran) or molecules that competitively bind to the anticoagulant agents (in the case of factor Xa inhibitors).

### Reversal of direct oral factor Xa inhibitors

Preclinical data suggest that rFVIIa and PCCs (activated and nonactivated) may be useful for the reversal of NOAC-induced anticoagulation. Experimental studies have demonstrated that the amelioration of coagulation parameters is associated with a beneficial effect on blood loss [33, 34]. In addition, a number of studies in human healthy subjects have revealed that the administration of PCC resulted in a correction of the prolonged prothrombin time and restored depressed thrombin generation after rivaroxaban treatment in a controlled trial in healthy human subjects. Similarly, a three-factor PCC (Profilnine®; Grifols Biologicals Inc., Los Angeles, CA, USA) was also evaluated for rivaroxaban reversal in a study in healthy volunteers and was shown capable of correcting some of the rivaroxaban-induced effects on coagulation parameters [35–37]. Recent studies confirmed these findings also at lower doses of PCCs [38, 39].

More specific reversal of anti-factor Xa agents can be achieved with new agents that competitively bind to the anti-factor Xa agents. Ciraparantag binds directly to the factor Xa agent (in particular edoxaban) via hydrogen bonds from or to various parts of the molecule [40, 41]. This antidote was shown to block the anticoagulant effect of edoxaban and restored the prothrombin time in vitro. Further development is ongoing. Similarly, andexanet-alfa is a recombinant protein analog of factor Xa that binds to factor Xa inhibitors but does not trigger prothrombotic activity. Andexanet virtually immediately reversed the anticoagulant activity of apixaban and rivaroxaban in healthy subjects without evidence of clinical toxic effects [42]. A clinical study in patients who present with bleeding while taking anti-factor Xa DOACs is ongoing.

Monitoring the reversal of the anticoagulant effect of factor Xa inhibitors is most simply done by measuring the prothrombin time, although there is some variability between prothrombin time reagents and for some agents the anti-factor Xa assay is more reliable [43]. Of note, the INR is not a suitable test to quantitate the (residual) anticoagulant effect by factor Xa agents.

### Reversal of direct oral thrombin inhibitors

The other group of DOACs directly targets thrombin (factor IIa) and is represented by dabigatran. Preclinical studies show variable effects for the efficacy of (activated) PCCs and factor VIIa to reverse the anticoagulant effect and to ameliorate experimental bleeding in animals exposed to dabigatran [33, 44, 45] Relatively high doses of PCCs, however, seem to have a reversing effect. Similarly, human volunteer studies show a limited effect of conventional doses of PCC to normalize coagulation parameters after ingestion of dabigatran [35, 37]. There are no systematic clinical trials investigating the effect of PCCs to reverse dabigatran-associated hemorrhage in clinical practice. However, some case series are helpful. The effectiveness of dabigatran-related bleeding management using a nonactivated four-factor (4F)-PCC (Octaplex^®^; Octapharma, Vienna, Austria) was recently evaluated by Diaz et al. [46]. Five patients receiving dabigatran, 76–88 years of age, were administered 4 F-PCC to manage dabigatran-related GI bleeding complications. Treatment with 4 F-PCC was able to adequately control bleeding in four of five patients; the fifth patient died of septic shock and coagulopathy secondary to severe hemorrhage. No thromboembolic events were reported in the next 6 months of follow-up in these patients. Interestingly, the authors also reported that, in the one patient presenting with a prolonged activated partial thromboplastin time (aPTT) as a result of dabigatran treatment, administration of 4 F-PCC was able to partially normalize this laboratory parameter. Similar clinical case reports have been reviewed elsewhere [47].

A direct reversing agent for dabigatran has been developed recently and is based on a Fab fragment of a monoclonal antibody (idaricuzimab) directly binding to dabigatran and eliminating its anticoagulant effect [48]. Experimental studies show a rapid and almost immediate reversal of dabigatran-induced anticoagulation [49, 50]. In healthy human subjects that were exposed to dabigatran, this antidote also showed a complete elimination of the anticoagulant effect of dabigatran [51]. In a clinical trial in 90 patients on dabigatran who had serious bleeding or required an urgent invasive procedure, idarucizumab completely reversed the anticoagulant effect of dabigatran almost instantaneously [52].

Monitoring of the anticoagulant effect of thrombin inhibitors in routine clinical practice is difficult. The aPTT is not very useful. An ecarin clotting time may be more accurate but is not readily available in most routine clinical settings. Most convenient and practically applicable for monitoring of the anticoagulant effect may be the diluted thrombin time, which needs to be standardized for the specific agent that was used [53].

## Conclusion

For the new generation of DOACs, several reversal strategies are under evaluation and specific antidotes or reversing agents are being developed, although most interventions need further evaluation in clinical trials. Such strategies can be used in case of serious hemorrhage complicating the use of DOACs or if a patient on DOACs needs to undergo an immediate invasive procedure.

## Abbreviations

aPTT, activated partial thromboplastin time; DOAC, direct oral anticoagulant; 4F, four-factor; INR, international normalized ratio; NOAC, novel oral anticoagulant; NSAID, nonsteroidal anti-inflammatory agent; PCC, prothrombin complex concentrate; RFVIIa, recombinant factor VIIa; VKA, vitamin K antagonist
